# Species identification and connectivity of marine amphipods in Canada’s three oceans

**DOI:** 10.1371/journal.pone.0197174

**Published:** 2018-05-23

**Authors:** Astrid Tempestini, Søren Rysgaard, France Dufresne

**Affiliations:** 1 Département de biologie et Québec Océan, Université du Québec à Rimouski, Rimouski, QC, Canada; 2 University of Manitoba, Centre for Earth Observation Science, Winnipeg, MB, Canada; 3 Greenland Institute of Natural Resources, Nuuk, Greenland; 4 Aarhus University, Arctic Research Centre, Aarhus, Denmark; 5 Département de biologie et Centre d’étude Nordique, Université du Québec à Rimouski, Rimouski, QC, Canada; Universita degli Studi della Tuscia, ITALY

## Abstract

Monitoring the distribution of marine biodiversity is a crucial step to better assess the impacts of global changes. Arctic marine fauna is dominated by amphipods in terms of abundance and biomass. These peracarids are an important marine order of crustaceans but the number of species found in the different Canadian oceans is currently unknown. Furthermore, most species are difficult to identify due to poor taxonomic descriptions and morphological convergence. The aim of this study was to assess the species diversity of marine amphipods in the three Canadian oceans using DNA barcoding. To do so, we produced a database of DNA barcodes of amphipods from the three Canadian Oceans publicly available from the BOLD website to which we added 310 new sequences from the Canadian Arctic Archipelago. We first delimited amphipod species based on barcode gap detection techniques and tree based method (bPTP) and then compared the composition of amphipods among the three oceans in order to assess the influence of past transarctic exchanges on Arctic diversity. Our analysis of 2309 sequences which represent more than 250 provisional species revealed a high connectivity between the Atlantic and Arctic Oceans. Our results also suggest that a single threshold to delimitate species is not suitable for amphipods. This study highlights the challenges involved in species delimitation and the need to obtain complete barcoding inventories in marine invertebrates.

## Introduction

Oceans cover most of the planet, but are still poorly known in terms of biological composition and species richness [1–4]. As climate changes and human pressures are growing, understanding the distribution of marine biodiversity is a crucial step towards an effective monitoring of marine ecosystems [2]. Nonetheless, numerous species of marine invertebrates are still awaiting taxonomic description [4]. The identification and estimation of species diversity based on a single genetic locus often appears the best option available for groups for which taxonomy is poor or inexistent [5, 6].

DNA barcoding proposed by [7] is a method for identifying unknown specimens to taxonomic entities based on sequence similarity of mitochondrial DNA (mtDNA) sequences [8, 9]. Inexact matches are either grouped with taxa already present in the database or identified as new based on whether they fall within a threshold of sequence similarity [8]. Therefore, delimiting species based on barcode requires setting a specific threshold beyond which two sequences will belong to different putative species referred as molecular operational taxonomic units (MOTUs). Several methods have been developed to delimitate species using single locus data such as the barcode gap detection [5, 10, 11] or based on phylogenetic trees [12, 13]. These methods have been successfully used in the exploration of biodiversity in insects [12], in crustaceans [10, 14, 15], in polychaetes [16], in echinoderms [17]. However, the delimitation of species based on a single locus can be problematic due to incomplete lineage sorting, heteroplasmy, introgression, young species showing no variation at COI or because it relies on taxonomic data [18–20]. Despite its constraints, DNA barcoding has become an important tool in biodiversity investigation leading to an increase amount of barcode data available.

Marine crustaceans are notoriously difficult to identify to the species level by traditional approaches due to their enormous morphological diversity and because morphological stasis is frequent in this group [21, 22]. Despite a decade of barcoding, only 7000 crustacean species have been barcoded out of a total of 67,000 described species so far, and it is estimated that there could be as many as 150,000 species world-wide [23]. In crustaceans, barcode gap detection as proposed by [7] has proven to be an efficient tool to discriminate species like marine crustaceans [22, 23], and more specifically in decapods [24] and amphipods [25, 26].

Amphipods are small crustaceans characterized by a direct development and weak active dispersal capabilities [27]. These characteristics favor cryptic speciation and endemism, and prevent widespread distribution as has been documented in isopods [28]. Despite their key role in the arctic food web [29], little is known about the biodiversity of marine amphipods. Amphipods dominate the arctic marine fauna in terms of abundance and biomass [30]. Despite the fact that Arctic regions are already impacted by global warming, the Arctic Ocean is one of the less understood region in the world and its marine biodiversity is one of the least characterized [30–33]. Arctic marine biodiversity has been shaped by the complex history and environment of this region. At least six openings of the Bering Strait since 5.5 million years ago have allowed trans-Arctic exchanges and invasions of the North Atlantic region by the North Pacific species [34–36]. Moreover, Quaternary glaciations have pushed some taxa out of the Arctic and recolonisation of the Arctic occurred from neighboring oceans during interglacial periods [17]. Several studies have documented these transarctic exchanges in molluscs [34, 37], in algae [38], in fishes [39, 40] and in polychaetes [16]. So, over millions of years, marine species have dispersed through the Arctic several times leading to a complex pattern of biodiversity [17].

In this study, we investigated the species delimitation of amphipods and the biodiversity of marine arctic amphipods using publicly available DNA barcodes to which we added new sequences for amphipods from the Canadian Arctic Archipelago. Our aims were 1) to test species delimitation using both distance-based and coalescent methods and 2) to assess large-scale connectivity of marine amphipods across the three Canadian oceans.

## Materials and methods

### Sampling and DNA amplification

Samples of Arctic amphipods were collected in 2011 in the Canadian Arctic Archipelago during the NCGS Amundsen expedition using vertical and/or horizontal nets with 200, 500 and 750 μm mesh size. Samples from Greenland were provided by the Greenlandic Institute of Natural Resources. Specimens from the southeastern Bering Sea were collected in 2012 during the Bering-Aleutian Salmon International Survey with a 500 μm mesh bongo net [41]. Specimens from Prince William Sound in the Gulf of Alaska were collected in 2013 with a 500 μm mesh ring net. All individuals were preserved in 95% ethanol. Samples were identified to the species level with appropriate taxonomic keys [42, 43] when possible. Description of samples with geographic cordinates can be found under the “CAAB” (Canadian Arctic Amphipods Barcodes) project available in BOLD (www.boldsystem.org).

The DNA of 374 samples was extracted using the E.Z.N.A tissue extraction kit (Omega-biotek), or the QuickExtract kit (Omegabiotek) following the manufacturer’s protocols. Individuals with a body size > 10 mm were extracted using one or two pereopods. Individuals with a size < 10 mm were used whole for the extraction. A 658 base pair (bp) fragment of the mitochondrial cytochrome *c* oxidase subunit I gene was amplified using the primer pair LCO1490/HCO2198 [44]. Polymerase chain reaction (PCR) was conducted as described in [45]: the reaction mix contained 1X PCR buffer, 2.2 mM MgCl_2_, 0.5 mM dNTPs, 0.4 μM of each primer, 1.5 U of Taq DNA polymerase (Life Technologies, Mississauga, ON, Canada), DNA template (around 40–80 ng), and water for a final volume of 25 μl. PCR were performed with an initial denaturation step of 3 min at 94 °C, followed by 5 cycles of 45 s at 94 °C, 45s at 46 °C, 45 s at 72 °C and 35 cycles of 45 s at 94 °C, 40 s at 51°C, 45s at 72 °C, and a final elongation step of 5 min at 72 °C. All PCR products were verified on a 1.5% agarose gel and direct-sequenced by Genome Quebec (McGill University, Montreal, Canada). Over the 374 individuals used, 310 individuals were successfully sequenced and their chromatograms were manually checked on MEGA7 [46].The presence of pseudogenes was assessed by translating sequences into amino acids. All sequences were deposited in the the project “CAAB” (“Canadian Arctic Amphipod Barcodes”) available in BOLD and in GenBank database under the accession numbers MH330696—MH331009.

### Dataset and molecular operational taxonomic units construction

Sequences found after searching in December 2017 for “Amphipoda” from Canada, Greenland and United States in the public data portal of BOLD conducted were combined to our data. All sequences were aligned using MUSCLE [47] available in the BOLD sequence analysis tools. The final dataset contained 2309 sequences from the three canadian oceans (S1 Table). As reliable MOTU depends on the accuracy of the MOTU retrieved with different methods [48, 49], we chose to used several gap discovery methods (Barcode Index Number, MOTHUR, ABGD) and coalescent process (bPTP) to find the number of MOTU present in our dataset.

#### Barcode Index Number (BIN)

The Barcode index number was constructed by first using a 2.2% p-distance threshold for clustering sequences and then each cluster was refined by the examination of the genetic divergence among neighbors [50]. Each cluster was described with a unique and specific identifier (e.g. Barcode Index Number or BIN), already available or newly created if the sequences are clustered in an unknown BIN [50]. Sequences were aligned using Kimura-2 parameters (K2P) [51], with MUSCLE [47]. All analyses were conducted in BOLD with sequence analysis tools.

#### MOTHUR

We used MOTHUR [52] to cluster our sequences into provisional species (referred as MOTU). In the literature, several thresholds are reported for delimiting species: a 3% threshold commonly used to define species [53], a 4% threshold proposed by [25], a 16% threshold for amphipods species proposed by [10]. Uncorrected pairwise distances were first computed for each threshold values and sequences were clustered into MOTU using the nearest neighbor method considering each gap. Briefly, this method allows the clustering of sequences in the same MOTU if they are at most X% distant from the most similar sequence in the MOTU.

In order to associate taxonomy to MOTUs, we created a molecular taxonomic database containing all amphipods identified to the species level and its barcode sequence. To do so, we selected all amphipod sequences from BOLD with the following criteria: taxonomic identification at least to the genus level and a COI sequence longer than 500 bp. All sequences were aligned with MAFFT version 7 web server [54] and trimmed to 501 bp. After the construction step, each MOTU was aligned to the reference database with a confidence threshold of 90%. In order to assess the accuracy of our molecular identification, we aligned 305 sequences of 43 taxonomic identified species to our taxonomic database. As no discrepancy was observed, we confirmed the validity for MOTU identification. All analyses were performed with MOTHUR [52].

### Automatic Barcode Gap Discovery (ABGD)

Since intra-specific divergences are smaller than inter-specific ones [55], a gap in the distribution of all pairwise distances can be identified using the Automatic Barcode Gap Discovery method available at www.abi.snv.jussieu.fr/public/abgd. This method is described in detail in [5]. Briefly, the data was first partitioned into a number of groups (i.e. species) such that the distance between two sequences taken from distinct groups was always larger than a given threshold distance and then appply recursively this procedure to get a better partitioning of the data into putative species [5]. We used the default value of 0.001 for the minimum intraspecific distance and 0.3 for the maximum intraspecific distance, with 10 steps and K2P distance. We explored the relative gap width (X) for X = 0.5 and X = 1. After the barcode gap discovery, sequences were clustered into MOTU based on the estimated threshold.

#### Coalescent approach

Poisson Tree Process is a model for delimiting species based on a rooted tree with branching events representing the number of substitutions [13]. As a large dataset is computer challenging, we selected unique sequences to reconstruct the tree and then performed the analysis. To reconstruct the tree, we rooted it with *Pandalus borealis* (Krøyer, 1838) (accession: KY018893.1). We selected the evolutionary model using the Bayesian Information Criterion (BIC) available at W-IQ-TREE [56]. We generated a phylogeny under the General Time Reversible model with empirical frequency, invariable site and under gamma rate (GTR+F+I+G4) using Bayesian inferences in MrBayes 3.2.6 [57] available at www.phylo.org, using two runs for 10,000,000 generations until convergence was observed. Trees were sampled every 10,000 generations and the first 25% of sampled tree were discarded as burn-in. The posterior probabilities (PP) were calculated with the 50% majority-rule consensus tree. We used the web version of bPTP (http://species.h-its.org/ptp/) to generate the species delimitation under a coalescent process. Analysis was conducted with 500,000 iterations of MCMC and 25% burn-in. We also removed the outgroup to improve the delimitation.

#### Diversity among the three canadian oceans

To provide an overview of the similarity in the MOTU composition among the three oceans, a Venn diagram was obtained for each threshold.

### Species threshold identification

As the thresholds proposed by [10] or by [25] were based on a single family of amphipods, we used the same method to identify a species threshold for all Amphipoda. To do so, we collected all amphipod sequences from BOLD (January, 2016). Among the 15516 records, all sequences without a taxonomic identification to the species level, with less than 500 bp or associated with pseudogenes were discarded. A total of 8471 sequences corresponding to 89 families were examined further. Families with less than 30 sequences or containing less than 3 species were also discarded from this dataset. The diversity assessments for the amphipods and for the most represented families were analysed from the data set with 3879 sequences from 272 species, 70 genera, and 10 families. After performing a first alignment with MAFFT [54], all sequences were trimmed to the same length of 501 bp. After this step, Kimura 2 parameters pairwise distances were computed at each taxonomic level intrafamily (F), intragenus (G) and intraspecies (S) in MEGA7 [46] and plotted by family using the boxplot representation available in R and described in [10]. Based on the ABGD, we estimated a general threshold to 7%. Three ranges of thresholds (3%, 7% and 16%) were plotted to see which one best discriminated the different amphipod species.

## Results

### Nucleotide diversity

Out of a total of 310 sequences obtained, 5 sequences contained stop codon and were removed from the analysis. All sequences were clustered into 28 MOTUs representing 26 BIN of which 3 were uniques. The mean GC content was 32.9%. Intraspecific K2P distance ranged from 0.6 to 18.07% and interspecific distance ranged from 1.3 to 27.3%.

**Fig 1:**
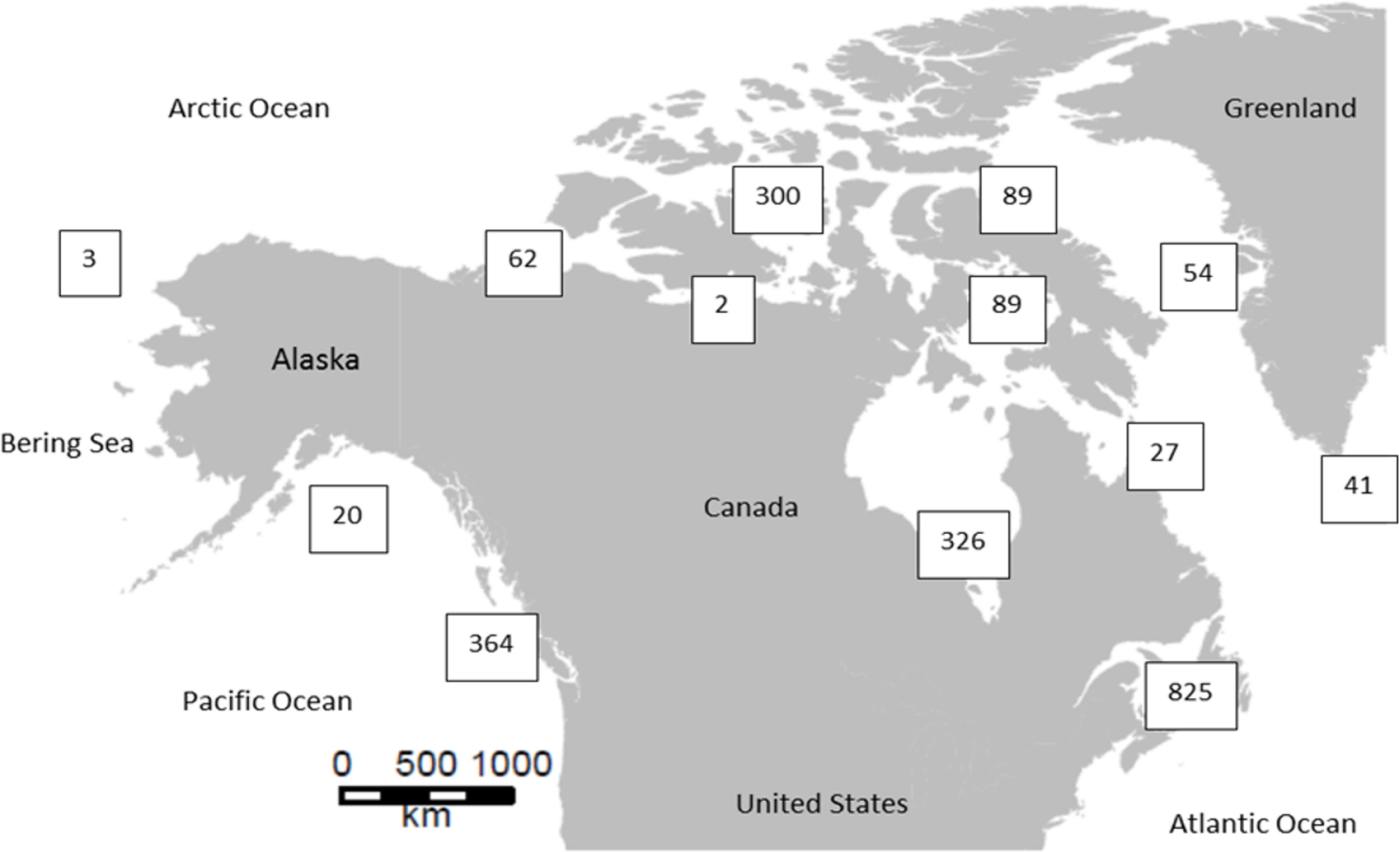
Map of the sampling location of each sequence used in this study.

The complete dataset consisted in 2309 sequences that were trimmed to the same length of 400 bp. Within the final alignement, 94 conserved sites and 277 parsimony informative sites were detected. The mean GC content was high (GC = 38.01%). Intraspecific K2P distance ranged from zero to 33.33% and interspecific distance ranged from 0.17 to 32.14%. There were 418 sequences from locations within the Pacific Ocean, 998 sequences from the Arctic region and 892 from the Atlantic (Fig 1).

### Gap distance based methods

#### BOLD

**Fig 2:**
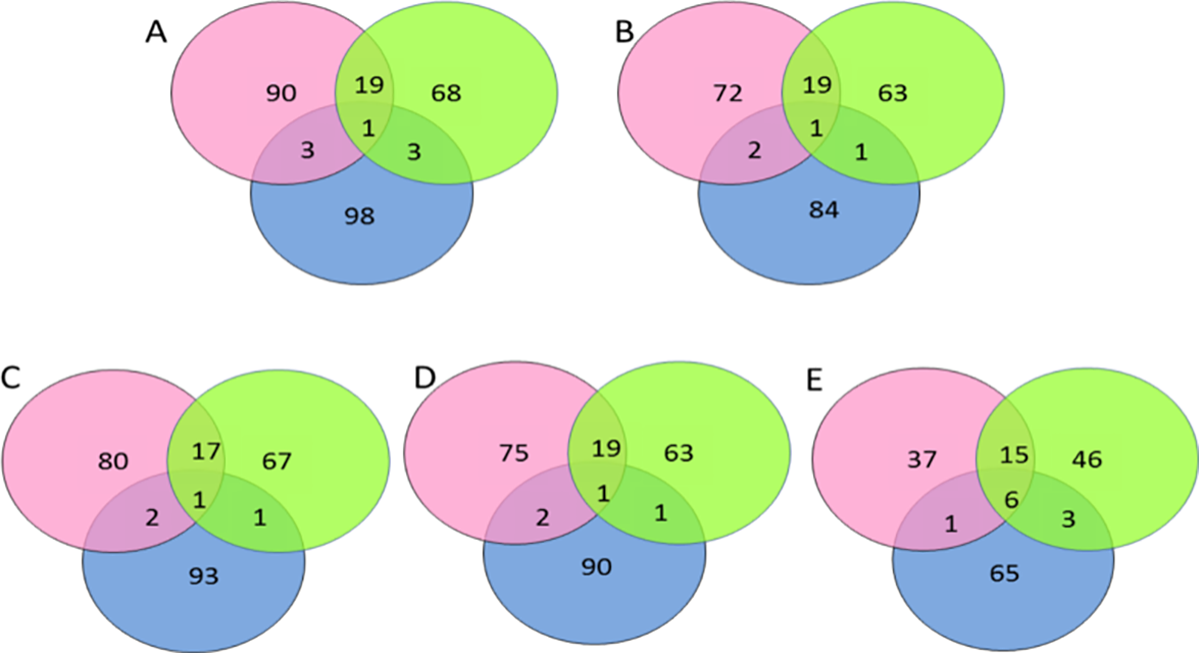
Venn diagram of shared MOTUs among the three oceans: Arctic (pink), Atlantic (green) and Pacific (blue). The number of MOTUs in each group and shared between groups is indicated. A: MOTUs were defined with a 3% threshold. B: MOTUs were defined with a 16% threshold. C: MOTUs were defined with a 4% threshold.

All 2309 sequences were grouped into 285 MOTUs and 263 BINs, of which 83 BINs were unique (Table 1, S2 and S3 Tables). Among these 285 MOTUs, 113 were from the Arctic, 91 were from the Atlantic and 105 were from the Pacific. Nineteen MOTUs were shared between the Arctic and the Atlantic, three were shared between the Pacific and the Atlantic, and three were shared between the Pacific and the Arctic. A single MOTU was shared among the three oceans (Fig 2A).

**Table 1:** Number of MOTUs found according to the method used.

Method	Threshold	Number of MOTUs
BOLD	BIN	263
	MOTU	285
ABGD	X = 0.5	242
	X = 1	242
MOTHUR	3%	261
	4%	251
	16%	173
bPTP		265–287

### Barcode gap detection with ABGD

The distribution of K2P genetic distances displayed two modes separated by a gap (‘barcode gap’) between 0.04 and 0.8. The ABGD method split 2309 sequences into 242 groups over a wide range of prior maximum divergence (P = 0.046416—P = 0.100000) after 20 partitions for X = 1 and after 23 partitions for X = 0.5 (Table 1, S1 Fig). All analyses produced a single group when P = 0.11. Among the 242 groups, 94 were from the Arctic, 84 from the Atlantic, and 88 from the Pacific (Fig 2B). One MOTU was shared between the Arctic and the Pacific, two between the Atlantic and the Pacific, and one among the three oceans. Nineteen MOTUs were shared between the Atlantic and the Arctic.

### MOTHUR

**Fig 3:**
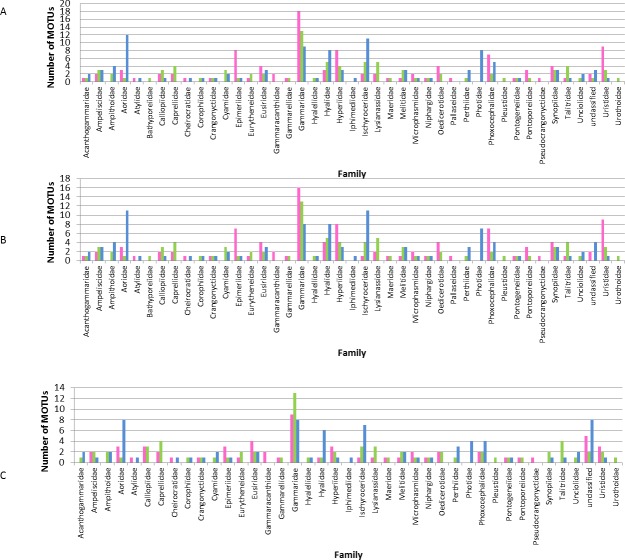
Distribution of MOTUs by family and ocean. **MOTUs are defined based on a threshold of A) 3%, B) 4%, C) 16%.** Atlantic MOTUs are represented in green, Pacific MOTUs in blue and Arctic MOTUs are in pink.

The 3% threshold allowed the identification of 261 MOTUs (Table 1), for which 100 were from the Arctic, 86 from the Atlantic, and 97 from the Pacific (Fig 2C). Eighteen MOTUs were shared between the Arctic and the Atlantic. In contrast, three MOTUs were shared between the Arctic and the Pacific and two between the Atlantic and the Pacific, of which one MOTU was common among the three oceans and identified as *Themisto libellula* (Lichtenstein in Mandt, 1822) (Fig 2C). A large proportion of MOTUs belonged to the Gammaridae, Ischyroceridae, Hyalidae and Aoridae families (Fig 3A). We were unable to identify 6 MOTUs to taxonomic level. At the species level, several species were found in the three oceans: *Ampelisca spinipes* (Boeck, 1861), *Aora gracialis* (Spence Bate, 1857), *Parhyale hawaiensis* (Dana, 1853), *Microphasma agassizi* (Woltereck, 1909), *Pontogeneia inermis* (Krøyer, 1838), *Tiron biocellata* (Barnard, 1962), *Weyprechtia pinguis* (Krøyer, 1838). As only one shared MOTU was detected between the three oceans, it suggests that these species consist of distinct MOTUs (Table 2).

**Table 2:** Species found in the three oceans.

	3%			4%			16%		
	Arctic	Atlantic	Pacific	Arctic	Atlantic	Pacific	Arctic	Atlantic	Pacific
*Ampelisca spinipes* (Boeck, 1861)	1	1	2	1	1	2	1	1	1
*Anonyx nugax* (Phipps, 1774)	0	0	0	0	0	0	1	1	1
*Aora gracilis* (Spence Bate, 1857)	1	1	7	1	1	6	1	1	3
*Microphasma agassizi* (Woltereck, 1909)	2	1	1	2	1	1	1	1	1
*Parhyale hawaiensis* (Dana, 1853)	1	2	1	2	2	1	0	0	0
*Parhyale sp*. *DP0017*	2	2	2	2	2	2	0	0	0
*Pontogeneia inermis* (Krøyer, 1838)	1	1	1	1	1	1	2	1	1
*Themisto libellula* (Lichtenstein in Mandt, 1822)	1	1	1	1	1	1	1	1	1
*Tiron biocellata* (Barnard, 1962)	4	3	3	4	3	3	1	1	1
*Weyprechtia pinguis* (Krøyer, 1838)	1	1	1	1	1	1	0	0	0

Numbers represent the number of MOTUs from each ocean identified to the species level. MOTUs are defined with a 3%, 4% and 16% threshold respectively.

Under the 4% threshold, 251 MOTUs were found for which 97 were in the Arctic, 84 in the Atlantic and 94 in the Pacific (Table 1, Fig 2D). Twenty MOTUs were shared between the Arctic and the Atlantic. Three MOTUs were shared between the Arctic and the Pacific, and two MOTUs between the Atlantic and the Pacific among which one MOTU is shared among the three oceans. Six MOTUs were shared among the three oceans and identified as *Ampelisca spinipes* (Boeck, 1861), *Aora gracilis* (Spence Bate, 1857), *Microphasma agassizi* (Woltereck, 1909), *Pontogeneia inermis* (Krøyer, 1838), *Parhyale hawaiensis* (Dana, 1853), *Themisto libellula* (Lichtenstein in Mandt, 1822), *Weyprechtia pinguis* (Krøyer, 1838), *Tiron biocellata* (Barnard, 1962) respectively (Table 2). Most MOTUs belong to the Gammaridae, Hyalidae and Ischyroceridae families (Fig 3B). Six MOTUs were not assigned to a taxonomic level.

Under the 16% threshold, 173 MOTUs were found, for which 59 were in the Arctic, 70 in the Atlantic and 75 in the Pacific (Table 1, Fig 2E). Twenty-one MOTUs were shared between the Arctic and the Atlantic. Three MOTUs were shared between the Arctic and the Pacific and nine MOTUs were shared between the Atlantic and the Pacific. Six MOTUs were shared among the three oceans and identified as *Ampelisca spinipes* (Boeck, 1861), *Aora gracilis* (Spence Bate, 1857), *Anonyx nugax* (Phipps, 1774), *Ischyrocerus anguipes* (Krøyer, 1838), *Microphasma agassizi* (Woltereck, 1909), *Pontogeneia inermis* (Krøyer, 1838), *Themisto libellula* (Lichtenstein in Mandt, 1822), respectively (Table 2). Most MOTUs belong to the Gammaridae family and the Aoridae family (Fig 3C). Only 11 MOTUs were not assigned to a taxonomic level.

### Tree based method

The tree-based bPTP analysis estimated the number of species between 265 and 287 with a mean of 275 species (S2 Fig, S4 Table) with high posterior probablilities (>0.5).

### Canadian Arctic diversity

**Fig 4:**
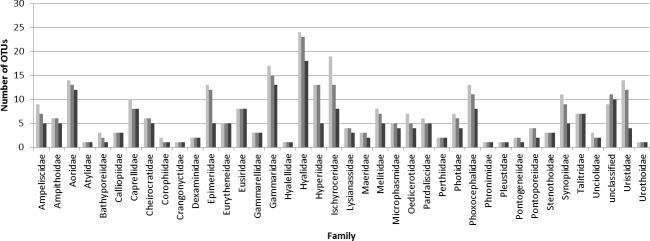
Number of MOTUs identified to the family level according to the species threshold used (3% light grey; 4% grey and 16% dark).

The family identification showed that not all the locations and the families were well sampled across the three Canadian oceans (Fig 3C). Eight families (Bathyporeiidae, Gammaracanthidae, Iphimedidae, Pallaseidae, Photidae, Pleustidae, Pseudocrangonyctidae, and Urothidae) were represented by a single MOTU in one location. The Gammaridae family had the highest number of MOTUs (Fig 4).

As expected, the number of MOTUs decreased with an increase of the threshold value used for all families (Fig 4). Six families were equally recovered (Bathyporeiidae, Corophidae, Iphimediidae, Pallaseidae, Pleustidae, Pseudocrangonyctidae) regardless of the threshold.

### Species threshold

**Fig 5:**
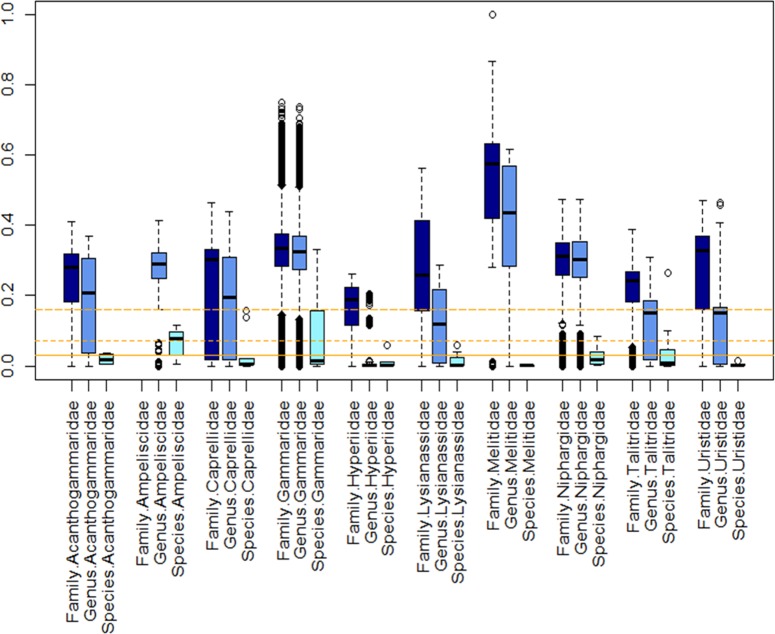
Boxplot distribution of the COI K2P distances for the 10 selected families representative of the Amphipoda order at the intraspecies (S–light blue), intragenus (G–blue) and intrafamily (S–dark blue) levels. Median (central bar), position of the upper and lower quartiles (central box) and extremes of the data (dots) are represented. Black lines represent different thresholds used for discriminating species: long dash = 0.16 substitution/site [10]; dashed line = 7% of divergence (this study) and solid line = 3% of divergence [53].

Divergences were estimated for the validated dataset from 3879 sequences representing 272 species, 79 genera, and 10 families. As expected, genetic divergence increased with taxonomic rank: a higher divergence was observed at the family level (K2P from 0 to 0.9), than at the genus level (K2P from 0 to 0.7), and the species level (K2P from 0 to 0.3). However, the threshold used to delimit species varied among families (Fig 5). The threshold proposed by [10] (0.16 substitution/site) discriminated well the Gammaridae species but not the other amphipods species from different genera. The lowest species divergence was observed in the Melitidae (0.001), the lowest genus and family divergences were observed in the Hyperiidae (0.26). The highest species (0.3) and genus (0.7) divergence was observed within the Gammaridae and the highest family divergence (0.9) was observed within the Melitidae.

## Discussion

Inventories of marine biodiversity are much needed in the context of global changes. A decade of DNA barcoding has resulted in the acceleration of species discovery and has helped to provide partial or complete inventories lists for selected taxa [23, 58]. The ability to assign species identities to DNA sequences depends on the availability of comprehensive DNA reference libraries such as BOLD [59]. The generation of these libraries represents an important task, in particular in some difficult acessible region such as the poles or the abyss where our knowledge on their biodiversity is still lacking [23]. With the increasing use of metabarcoding approach, the need for complete library references will become essential to allow an easier investigation of species richness and to monitor the faith of the biodiversity in an area undergoing global changes such as the Arctic ocean [14].

Our study provides a useful example of how one can retrieve information from BOLD database to produce an exhaustive first step inventory of specific animal groups or specific regions that are otherwise lost among the different publicly available projects. We showed that widespread species are in fact composed of different MOTUs, suggesting the presence of cryptic species. This implies that a careful look at the taxonomic keys is needed for amphipods. Moreover, we found that using a single threshold for species delimitation for different families of amphipods is not always accurate.

### MOTU delimitation

The number of MOTU retrieved varied between 173 (MOTHUR-16% threshold) and 285 (BOLD). Excluding the highest threshold used in MOTHUR, ABGD retrieved the lowest number of MOTUs. Some discripancy have been noticed among methods. For example, da Silva *et al*. [60] noticed that ABGD and PTP produce lower estimates of snapper species diversity (based on the COI) than other methods. The relative performance of species delimitation methods has been examined in other studies [61]. The results produced by AGBD vary according to the metrics used and with the number of individuals per species and might be useless in the exploration of species diversity in poorly known groups [10, 61, 62]. Several authors emphasize the importance of combining several methods in species delimitation [6]. Defining species based on genetic data alone might be limiting and additional characters such as life history traits and geographic ditribution are also of interest for species description [6, 63].

Delimiting species based on a single mitochondrial fragment can introduce some bias. First, the DNA barcoding protocol has different steps that can introduce some errors that can lead to misidentification of the specimen [22]. Second, incomplete lineage sorting or homoplasy can contribute to an overlap between the intra- and interspecific distances leading to difficulties in the identification of the barcode gap [19]. “COI like” sequences can contribute to increase the threshold estimate as it has been investigated in crustacean [64].

We also conducted our species clustering analyses using a tree-based method. We found around 270 species which is less than the number of MOTUs found with BOLD (285) and less than the number of MOTUs found with ABGD (242). It is well known that tree based methods like GMYC or PTP are sensitive to the genealogy of a particular locus, wich can be discordant with the true species tree [65, 66]. Moreover, singletons can also represent difficulties [67, 68].

### Species threshold

Delimiting species relies on a threshold over which species belong to the same or to two different species. The 3% of divergence [53] is a commonly used threshold in the literature. Other studies report that a 16% threshold was more suitable for delimiting amphipod species like Gammaridae or Niphargidae [10, 69, 70, 71]. On the contrary, lower value for threshold has been also reported in other family of amphipods like in Talitridae (8% -17%) [71] or in Hyallelidae (4%) [72]. By including ten amphipod families, we have showed that the use of 16% is appropriate for the discrimination of Gammaridaea species but is too high to discriminate species from other families. Based on the interspecific distance distribution among amphipod families, we found that this threshold can discriminate most amphipod species but is not always suitable.

Based on the automatic barcode gap detection, we estimated a threshold of 7% for discriminating amphipod species, which is intermediate between the previous thresholds proposed. This threshold seems appropriate for the majority of amphipod species but again, based on diverse family distances distribution, this threshold will underestimate the number of Gammaridae or Talitridae species. Instead, we found that for discriminating amphipod species, a threshold specific to each family will be more appropriate. However, we should emphasize that not all amphipod families are equally studied and less than 10 sequences are not enough to estimate intraspecific distances [19, 67]. For example, Gammaridae is one of the best studied amphipod family for which a large amount of molecular data is available but other amphipod families such as Hyperiidae have not received the same attention. Here, we focused on 10 amphipod families that represent less than a quarter of all amphipod families [43]. Nevertheless our analysis provides the first molecular attempt to determine species threshold in amphipods. Additional barcoding data is needed on the other amphipod families for helping to refine our species threshold and to determine factors (e.g. benthic vs pelagic lifestyle, life history traits, speciation rates, effective population size, etc…) responsible for the large variation in sequence divergence among different crustacean families.

### Arctic diversity

Our study, based on the analysis of more than 2300 sequences distributed throughout the three Canadian oceans, indicates the presence of at least 250 provisional amphipod species. We found 100 putative Arctic species representing circa 85% of the known amphipods inventory in the Arctic (from the Chukchi Sea) [73]. We recovered more putative species in the Atlantic and less in the Pacific. Marine Arctic fauna is mostly derived from recent and repeated colonisations from both Pacific and Atlantic species after Pleistocene glaciation events or multiple Bering Strait openings [reviewed in 17 and 34]. Most studies on trans-arctic interchanges have reported a Pacific origin of the invasion [34, 38, 39, 74–78]. Regarless of the threshold used, our results indicate a higher similarity between Arctic and Atlantic oceans (>15 MOTUs shared) than between the Arctic and the Pacific (one MOTU shared). Similarity between Arctic and North Atlantic fauna has also been reported in polychaetes [16] or in bryozoans [79], suggesting that the Atlantic Ocean contributed significantly to the recolonization of the Arctic. In addition, the Pacific harbors the highest number of MOTUs compared to the number of sequences available (418), corroborating a higher diversity of this ocean compared to others [16, 34]. The limited number of shared MOTUs between the Arctic and Pacific oceans suggests the presence of a barrier restricting exchanges between these oceans. Moreover, the fact that the retrieved Pacific MOTUs were not found elsewhere confirms the isolation of Pacific taxa from colder Arctic waters. In copepods [80, 81] and in amphipods [82], isolation between Pacific and Arctic populations has been documented. The cold temperature of the Arctic waters has likely impeded the survival and reproduction of North Pacific species. Global warming induced changes in the Arctic ocean leading to less inhospitable barriers for Pacific species and promoting interchanges between Pacific and Atlantic oceans as suggested by recent models [83]. Therefore, further sampling of the Pacific region is needed to confirm this isolation as our results might be biased by differential sampling efforts between the Pacific and the Atlantic oceans.

We found a relatively higher proportion of benthic species belonging to the intertidal or infralittoral family of Gammaridae, Hyalidae and Ischyroceridae [84, 85] than pelagic species. In the unique pelagic amphipod family (e.g. Hyperiidae), we recovered 12 MOTUs of which the majority (8 MOTUs) were Arctic. In the Eurasian arctic waters inventory, the presence of eight Hyperiidae species were recorded [73]. This result suggests that pelagic diversity is not well known maybe due to sampling difficulties; further efforts are needed to better characterized ocean diversity. As sampling in the Arctic is quite challenging, it is most likely that rare taxa were not included in our analyses (S3 Fig–S5 Fig). Although, our study does not include depth information, it will also be interesting to investigate the diversity of MOTUs among oceans according to depth to get a more precise picture of the marine biodiversity.

However we were not able to identify all MOTUs to the species level. Several explanations can be considered. First, during the taxonomic reference creation, we discarded more than half of the sequences due to pseudogenes or short sequences. Secondly, the 15 516 sequences of amphipods available in BOLD correspond to 1 514 species which is under the number of amphipods estimates [4]. Despite these constraints, barcoding techniques have provided useful information on the amphipod biodiversity in the three Canadian oceans. This approach can benefit the study of oceanic Arctic region which is one of the least studied [3, 23, 33]. Moreover, we also showed that widespread marine amphipods species are composed of different MOTUs, suggesting an underestimated diversity. A large number of studies have revealed the presence of cryptic species complex in marine invertebrates[16, 55, 86–88], suggesting the utility of combining different types of data (e.g. molecular and morphological) to identify species.

## Conclusions

Our analyses have contributed to the assessment of marine arctic amphipod biodiversity in revealing potential cryptic species, in showing the sharing of MOTUs between the Arctic and the Atlantic amphipods and in the isolation of Arctic amphipods from those of the Pacific. Moreover, thanks to the increasing barcode data available in amphipods, we were able to show that threshold value for species identification in amphipods needs to be estimated for each family. It has become evident that species definition should not be restricted to a COI sequence but should include additional information such as ecological niche. Pursuing arctic amphipods studies with DNA barcodes will ultimately lead to a better understanding of marine biodiversity and the mechanisms of speciation in marine environments. With arctic waters already showing the presence of temperate invaders [89], there is an urgency to complete this task.

## Supporting information

S1 Table Dataset used in this study. (XLSX)

S2 Table Common BIN identified in this study. (XLSX)

S3 Table Unique BIN identified in this study. (XLSX)

S4 Table List of potential species found with bPTP. (XLSX)

S1 Fig The automatic partition results by ABGD with two X-values. In red, X = 0.5. In green, X = 1.
(DOCX)

S2 Fig PTP species delimitation tree. Red clades represent putative species. bPTP analyses resulted in an identical topology and putative species.
(EPS)

S3 Fig Rarefaction curve showing the number of MOTUs found in each ocean according the the number of sequence sampled. MOTUs are defined at 3% threshold.
(DOCX)

S4 Fig Rarefaction curve showing the number of MOTUs found in each ocean according the the number of sequence sampled. MOTUs are defined at 4% threshold.
(DOCX)

S5 Fig Rarefaction curve showing the number of MOTUs found in each ocean according the the number of sequence sampled. MOTUs are defined at 16% threshold.
(DOCX)
