# Supplementary material for: Species identification and connectivity of marine amphipods in Canada’s three oceans
Source: PLoS One. 2018 May 23;13(5):e0197174. doi: 10.1371/journal.pone.0197174 (PMC5965885; doi:10.1371/journal.pone.0197174)
Supplement: S1 Fig — In red, X = 0.5. In green, X = 1. (DOCX) [file pone.0197174.s005.docx]

**S1 Fig. The automatic partition results by ABGD with two X-values.** In red, X=0.5. In green, X= 1.
