# Supplementary material for: Species identification and connectivity of marine amphipods in Canada’s three oceans
Source: PLoS One. 2018 May 23;13(5):e0197174. doi: 10.1371/journal.pone.0197174 (PMC5965885; doi:10.1371/journal.pone.0197174)
Supplement: S4 Fig — MOTUs are defined at 4% threshold. (DOCX) [file pone.0197174.s008.docx]

S4 Fig. Rarefaction curve showing the number of OTUs found in each ocean according the the number of sequence sampled. OTUs are defined at 4% threshold.
